# Production of highly knotted DNA by means of cosmid circularization inside phage capsids

**DOI:** 10.1186/1472-6750-7-94

**Published:** 2007-12-21

**Authors:** Sonia Trigueros, Joaquim Roca

**Affiliations:** 1 Institut de Biologia Molecular de Barcelona, CSIC, Jordi Girona 18-26, 08034 Barcelona, Spain; 2 Department of Biochemistry, University of Oxford, Oxford OX1 3QU, UK

## Abstract

**Background:**

The formation of DNA knots is common during biological transactions. Yet, functional implications of knotted DNA are not fully understood. Moreover, potential applications of DNA molecules condensed by means of knotting remain to be explored. A convenient method to produce abundant highly knotted DNA would be highly valuable for these studies.

**Results:**

We had previously shown that circularization of the 11.2 kb linear DNA of phage P4 inside its viral capsid generates complex knots by the effect of confinement. We demonstrate here that this mechanism is not restricted to the viral genome. We constructed DNA cosmids as small as 5 kb and introduced them inside P4 capsids. Such cosmids were then recovered as a complex mixture of highly knotted DNA circles. Over 250 μg of knotted cosmid were typically obtained from 1 liter of bacterial culture.

**Conclusion:**

With this biological system, DNA molecules of varying length and sequence can be shaped into very complex and heterogeneous knotted forms. These molecules can be produced in preparative amounts suitable for systematic studies and applications.

## Background

The occurrence of knotted DNA molecules is common in biological systems. DNA knots were first observed *in vitro *near four decades ago in single-stranded DNA rings incubated with bacterial topoisomerase I [1] and in double-stranded DNA molecules extracted from phage P4 [2,3]. Later on, DNA knots were discovered in plasmids undergoing transcription and replication in bacteria with deficient topoisomerase activity [4,5]. Along these findings, the development of electron microscopy for RecA-coated DNA molecules [6,7] and of high resolution agarose gel electrophoresis [8-11] allowed the identification of numerous knot types shaped into DNA. Knot analyses with these techniques had been very useful to infer physical properties of double stranded DNA. For instance, the effective diameter of duplex DNA was determined from the fraction of knotted circles found after the circularization of linear DNA molecules that joined cohesive ends in free solution [12,13]. Also, the specific knot types produced when DNA recombinases [14,15] and topoisomerases [16,17] act on circular DNA was informative to reconstruct the architecture of these protein-DNA ensembles. However, the biological relevance and potential applications of knotted DNA molecules remain to be explored. DNA knots could play a role in the high order organization of chromosomes, yet they should not interfere DNA replication and transcription. Knotted DNA molecules will be also useful to further investigate biophysical properties of constrained DNA, as well as the activity of topoisomerases, recombinases and other DNA interacting ensembles. To facilitate these studies, a method to produce abundant DNA knots would be highly valuable. Here we developed the P4 phage system as a convenient source of knotted DNA.

The mechanisms of genome propagation by phage P4 had been elucidated thanks to the studies of Richard Calendar and colleagues [18]. P4 is a satellite phage that needs the helper prophage P2 for proliferation. When P4 infects a bacterial host lysogenic for P2, the 11.2 kb P4 genome is injected as a linear double-stranded DNA that quickly circularizes by ligation of its terminal cohesive ends [19]. P4 uses then the machinery of P2 to amplify and package its own genome. Contrary to phage lambda, which packages its genome from replicated linear DNA multimers, P4 uses covalently closed DNA circles as a preferred substrate for DNA packaging [20,21]. Each P4 DNA circle is cleaved at the 55-bp *cos *sequence to produce a linear molecule with the 19-bp cohesive ends, which is actively threaded into a P4 capsid [22]. Newly made P4 phages lead then to bacterial lysis and start a new infective cycle. These studies soon conducted to the discovery that a large fraction of DNA molecules extracted from phage P4 were highly knotted nicked DNA circles [2,3]. Formation of these knots was found enhanced in P4 phage derivatives with genome deletions [23] and in tailless-mutants [24]. Subsequent research indicated that DNA knot formation was caused by the premature circularization of the P4 genome inside the small volume of the phage capsid [25].

**Figure 1:**
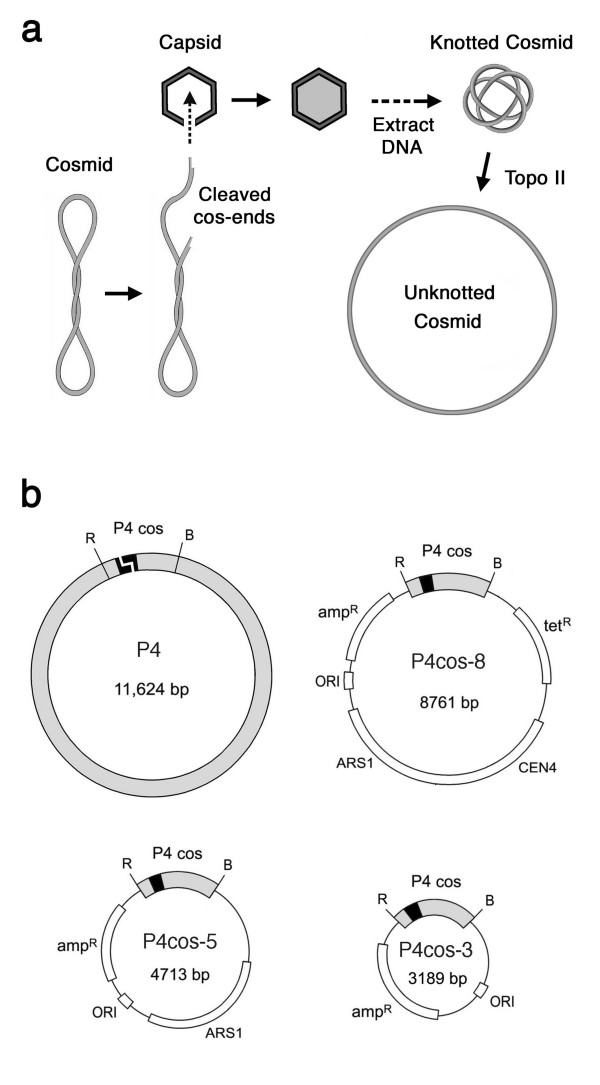
**P4 phage cosmids**. (**a**) A cosmid containing the *cos *sequence of phage P4 is cleaved and threaded into a viral capsid in the course of a bacterial phage infection. Circularization of the cosmid by its cohesive ends inside the phage capsid produce a highly knotted DNA circle. DNA knots are removed by topoisomerase II. (**b**) Scheme of circularized P4 DNA and of cosmids P4cos-8, P4cos-5 and P4cos-3, in which an *EcoRI*-*BamH1 *1189 bp fragment containing the P4-*cos *sequence is inserted.

P4 DNA knots are complex, heterogeneous and can be purified in preparative amounts [25]. Because DNA circularization results from non-covalent joining of 19-bp cohesive ends [26], the knots can be directly analyzed without requiring enzymatic nicking. Otherwise the knots can be readily converted into covalently closed DNA circles by a brief reaction with DNA ligase. However, one drawback of this natural system is that knot formation is restricted to the viral DNA. Hence, we asked whether other DNA molecules, of length and sequence different than P4 DNA, could be packaged inside P4 capsids and recovered also as highly knotted forms. Accordingly, we envisaged that a bacterial plasmid containing the P4 *cos *sequence (i.e. a P4 cosmid) could be cleaved and threaded into a viral capsid in the course of a bacterial infection by phage P4 (Figure 1a). Therefore, we constructed different P4 cosmids and introduced them in bacteria lysogenic for P2. These bacteria were then infected with P4 phage and the DNA in newly formed viral particles was analysed. We found that cosmids as small as 5 kb were packaged inside P4 capsids. More interestingly, as well P4 DNA, such cosmids were recovered in the form of highly knotted DNA circles.

## Results and Discussion

### Construction of DNA cosmids for *in vivo *packaging in phage P4

Linear P4 DNA molecules were converted into covalently closed DNA circles by annealing their terminal 19-bp cohesive ends and sealing them with DNA ligase. Digestion with restriction endonucleases *EcoR1 *and *BamH1 *generated an 1189 bp fragment that contained the joined P4 *cos *sequences. This fragment was inserted in several plasmids to generate P4 cosmids of different length, such as P4cos-8, P4cos-5 and P4cos-3 (Figure 1b). P4 cosmids were introduced in the *E. coli *strain C-1895 and transformants were selected by ampicillin resistance. Bacteria harbouring cosmids were infected with phage P4 *vir1 del22*, which has a 1.2 kb genome deletion that enhances its knotting probability. Replicated phages released upon bacterial lysis were purified. The amounts of phage particles containing P4 DNA or cosmid DNA were then estimated by their capacity for infecting (bacterial lysis) or for delivering the cosmid (ampicillin resistance) to new host bacteria, respectively. Relative to the amount of infective P4 phages recovered, the fraction of P4 particles able to deliver the cosmid to new *E. coli *cells was 3% for P4cos-8, 12% for P4cos-5 and <0.1% for P4cos-3. Therefore, P4cos-8 and P4cos-5, but not P4cos-3, appeared to be packaged in phage particles. Similar experiments with other DNA constructs indicated that the minimum cosmid size successfully packaged and delivered by P4 particles is about 5 kb. We ignore why shorter cosmids were not transferred by phage P4. A minimum DNA length is known to be required for efficient transduction in other phage systems [27]. Too short DNA molecules may preclude a correct assembly of the phage particle or may produce insufficient ejection forces to deliver the DNA [28].

### Knotting probability of cosmids extracted from P4 viral particles

**Figure 2:**
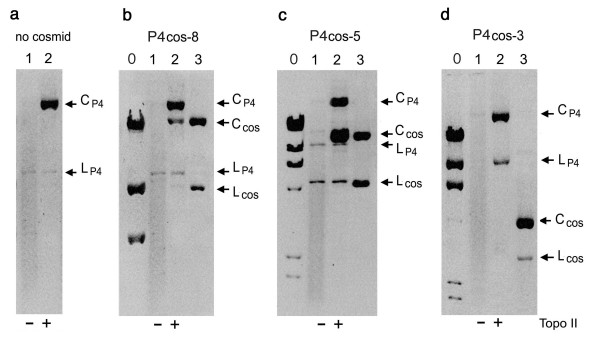
**Packaging efficiency and knotting probability of cosmid DNA in P4 phage particles**. DNA was extracted from either no cosmid (**a**), P4cos-8 (**b**), P4cos-5 (**c**) and P4cos-3 (**d**). Samples were loaded in the gel before and after unknotting the DNA with topoisomerase II (lanes 1 and 2, respectivelly, in a-d). Gel electrophoresis were done in 0.6% agarose gels with TBE buffer (1.4 volt/cm × 30 hours) in a and b; and in 0.8% agarose gels with TBE buffer (1.6 volt/cm × 20 hours) in c and d. Lambda DNA digested with HindIII was loaded in lane 0 in b, c, d. Nicked-circular (C_COS_) and linear (L_COS_) forms of each corresponding cosmid, produced by restriction endonuclease treatment of the supercoiled plasmids, were loaded as markers in lane 3 in b, c, d. Positions of the unknotted nicked-circle (C_P4_) and the linear form (L_P4_) of P4 DNA are indicated.

To determine the knotting probability of packaged cosmids, DNA extracted from the phage particles was examined by gel electrophoresis. When P4 phages were amplified in bacteria harbouring no cosmid, only P4 DNA was recovered. Because the knot distribution of P4 DNA is broad and complex [11], it produces a long smear of DNA in the gel (Figure 2a, lane 1). Incubation with topoisomerase II converted these knotted forms into the unknotted nicked DNA circle, easily identified as a single gel band (Figure 2a, lane 2). When P4 phages were amplified in bacteria harbouring P4cos-8, extracted DNA produced also a smear of knotted forms (Figure 2b, lane 1). Following topoisomerase II treatment, however, knotted molecules were converted into two gel bands of unknotted nicked DNA circles (Figure 2b, lane 2). One band was P4 *vir1 del22 *DNA (10 kb) and the other band was the P4cos-8 cosmid (8.7 kb), thus indicating that originally (inside the phage capsids) both types of DNA were knotted circles. Gel band quantifications indicated that nearly 20% of the viral particles had packaged P4cos-8 and that its knotting probability was >95%. Note that little unknotted cosmid is discernible before DNA unknotting by topoisomerase II.

Similar experiments were done for cosmids P4cos-5 and P4cos-3 (Figure 2c and 2d, respectively). When P4 phages were amplified in bacteria harbouring P4cos-5, over 60% of resulting viral particles packaged the cosmid, which had knotting probability >95%. When P4 phages were amplified in bacteria harbouring P4cos-3, the cosmid was not recovered in the viral particles. Therefore, as predicted by the transduction assays, P4cos-3 fails to be packaged into P4 particles. Larger cosmids, however, are efficiently packaged *in vivo *like P4 DNA and mostly recovered as knotted circles. Yet, notice that the fraction of cosmid DNA recovered in phage particles does not agree with the fraction of P4 particles able to transduce ampicillin resistance. This discrepancy likely reflects that only phage particles containing no circularized DNA, that is unknotted molecules, are able to deliver the cosmid into new host bacteria. Accordingly, we had also observed that a bacterial infection with native phage P4 (knotting probability < 50%) produces a fraction of infective particles larger than an infection with P4 *vir1 del22 *(knotting probability > 90%) [25]. Possibly, only linear DNA molecules with one of its cohesive ends interacting with the tail knob of the phage can be injected into bacteria [29].

### Knot distribution of P4 cosmids

**Figure 3:**
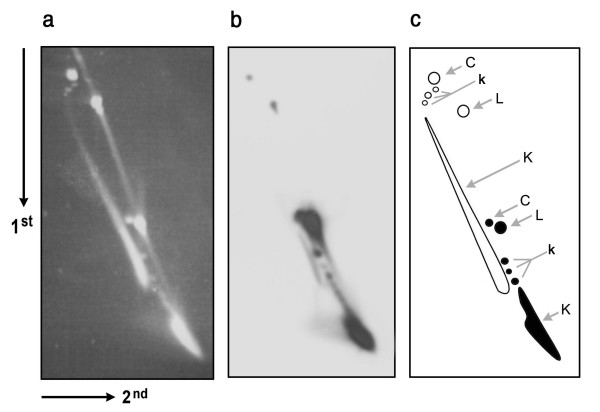
**Analysis of cosmid knots by two-dimensional gel electrophoresis**. DNA extracted from P4 *vir1 del22 *viral particles, which were amplified in bacteria that harboured P4cos-5, was electrophoresed in a gel slab of 0.4% agarose equilibrated with TBE buffer. The first gel dimension (top to bottom) was at 0.8 V/cm for 36 h at room temperature. The second gel dimension (left to right) was done in the same electrophoresis buffer at 3.4 V/cm for 4 h at room temperature. (**a**) Ethidium bromide staining of the gel. (**b**) The gel blotted to a nylon membrane and probed for P4cos-5. (**c**) Scheme showing the gel positions of linear molecules (L), unknotted circles (C), and knotted circles (K) of P4 DNA (white) and P4cos-5 (black). Dimeric P4cos-5 molecules (2 × 4.7 Kb) would ran nearly as P4 DNA (10 kb).

Knotted DNA extracted from viral particles containing P4cos-5 was analysed by two-dimensional agarose gel electrophoresis (Figure 3). The first dimension separated knotted circles with the same size according to their minimal crossing number [8]. The second gel dimension was at high voltage and segregated knotted circles from linear DNA molecules [30]. As expected, ethidium staining of the gel (Figure 3a) revealed two distributions of knotted molecules: One corresponded to P4 *vir1 del22 *DNA; and the other corresponded to P4cos-5, as confirmed by probing the gel-blot with cosmid DNA sequences (Figure 3b). As illustrated in Figure 3c, in both distributions, knot populations of low crossing number were discernible as individual gel bands (k) migrating slightly faster than the corresponding unknotted circles (C). More complex knots had higher gel velocity and form a dense tail of DNA (K). Note also that dimeric forms of the cosmid were negligible in the probed gel blot. Since a dimer of P4cos-5 (2 × 4.7 Kb) would ran almost overlapping P4 *vir1 del22 *(10 kb), faint signals observed in this region may even result from unspecific hybridization with P4 DNA (Figure 3b). Therefore, although each phage particle could hold two lengths of P4cos-5, apparently only one cosmid length was usually packaged and circularized inside each viral capsid. Yet, it cannot be formally excluded that that two cosmid molecules were packaged and circularized as monomers. This possibility would imply a mechanism that precludes inter-molecular annealing of their cohesive ends inside the capsid.

**Figure 4:**
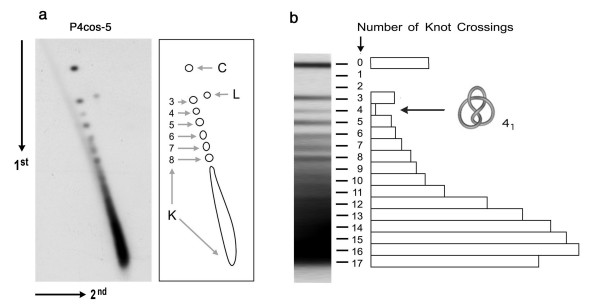
**Knot distribution of cosmid DNA**. (**a**) P4 viral particles containing P4cos-5 were purified by equilibrium density centrifugation. Extracted DNA was then electrophoresed in a 0.8% agarose gel equilibrated with TBE. The first dimension (top to bottom) was at 0.8 V/cm for 40 h at room temperature. The second dimension (left to right) was done in the same buffer at 3.4 V/cm for 4 h at room temperature. The gel blot was probed for P4cos-5. Positions of linear molecules (L), unknotted circles (C), individual knot populations containing three to eight crossings (3–8), and the tail of more complex knots (K) are indicated. (**b**) The electrophoresis velocity at low voltage (first gel dimension) is projected to estimate the complexity of the knot distribution. The histogram plots relative amounts of knot populations of increasing complexity by considering a linear relation between their gel velocity and their crossing number. The position and ideal configuration of the achiral knot of four crossings (4_1_) is indicated.

Next, to obtain knotted cosmids free from contaminating P4 DNA, we took advantage of the equilibrium density centrifugation of the viral particles in cesium chloride. Particles containing cosmid were less dense than those containing P4 DNA and, therefore, they equilibrated in a separated density band. Phages containing the cosmid were so purified by centrifugation. Gel electrophoresis conditions were then adjusted to display the cosmid knots with higher resolution (Figure 4a). Individual gel bands corresponding to the linear form (L), the unknotted circle (C), and to individual knot populations from 3 to 8 crossings were now discernible. More complex knots remained embedded in a dense tail (K) of higher gel velocity, which could not be further resolved with longer runs or with other electrophoresis buffers. This difficulty in separating the higher complexity forms reflected a large heterogeneity of knots types. In low voltage conditions the migration of different knots is not anymore the function of their minimal crossing number but a function of the overall compaction imposed by different knot types, which in turn is proportional to the average crossing number of ideal geometric representation of a given knot type [9,31]. Regarding the minimal crossing number of analysed knots we estimate that more abundant knots have the minimal crossing number of about 13 while the fastest migrating forms have the minimal crossing number of about 17 (Figure 4b). Remarkably, the mean number of topological crossings per unit length of the cosmid knots (13/4.4) was comparable to that reported for P4 DNA knots (26.4/11.2) [25]. Moreover, several landmarks of the cosmid knot distribution were analogous to those observed in P4 DNA [32]. First, there was a scarcity of the achiral knot of 4 crossings (4_1_) relative to knots of 3, 5, 6, 7 and 8 crossings. Second, there was a single band for the two possible knot types of 5 crossings (5_1 _and 5_2_). Longer gel runs identified this band as 5_1_, which has lower average crossing number than 5_2_. As proposed for the P4 genome [32], these traits suggest that cosmid DNA is highly writhed inside the phage capsid, so the formation of specific knot types is favoured [33].

## Conclusion

Bacteria lysogenic for helper prophage P2 can be transformed with plasmids (5 to 10 kb) containing the *cos *sites of the P4 phage (P4 cosmids). Subsequent infection with satellite P4 phage results in bacterial lysis with the release of a substantial fraction of viral particles containing a P4 cosmid. Such cosmids are then recovered in the form of highly knotted DNA circles (knotting probability > 95%). The distribution of knot types is very similar to that previously reported for P4 DNA. Therefore, the packaging and knotting processes of DNA inside P4 phage capsids are not exclusive properties of the viral genome. These findings may facilitate future studies on the structural properties of the P4 phage system and on the folding of DNA under the effects of confinement. Yet, a more immediate application of our results is the opportunity to generate complex knot distributions in DNA molecules of length and sequence different than the P4 genome. Usually, 250 μg of knotted cosmid are obtained from 1 liter of bacterial culture. Such DNA molecules are suitable for systematic studies on topoisomerase activities, biophysical properties of constrained DNA, and the effect of DNA knotting on biological transactions.

## Methods

### Plasmids, bacteriophages and bacterial strains

Plasmids used to construct P4 cosmids carried the pMB1 origin of replication and the *bla *(amp^R^) marker from pBR322 for selection by ampicillin. Bacteriophage P4 *vir1 del22*, which carries a 1.2 kb DNA deletion; and the *E. coli *strain C-1895, which is lysogenic for the helper prophage P2, were provided by Richard Calendar (University of California, Berkeley).

### *In vivo *packaging of P4 cosmids inside phage particles

Cosmids P4cos-8, P4cos-5 and P4cos-3 were transferred into *E. coli *C-1895 and resulting transformants were selected by ampicillin resistance. Transformants harbouring P4cos-8, P4cos-5, or P4cos-3 were infected with P4 *vir1 del22 *as follows: Individual bacterial colonies were grown overnight in 15 ml LB a 37°C without aeration. P4 phages (about 10^8 ^infective units) and CaCl_2 _(to a final concentration of 1 mM) were then added to the culture. Following 10 min incubation at 37°C, the infected culture was diluted into 400 ml LB (supplemented with 0.1% Glucose, 1.6 mM MgCl_2_, 0.5 mM CaCl_2_) and incubated at 37°C with fast shaking and good aeration. When bacterial lysis began (OD A_600 _drops usually 2–3 hours post-infection), EGTA pH 8.8 was added to a final concentration 5 mM and the incubation continued for 1 hour. Bacterial debris were removed by centrifugation (6000 × g for 15 min at 20°C). PEG 8000 and NaCl were dissolved by stirring in the supernatant fraction to a final concentration (w/v) of 8% and 2.5%, respectively. After 2–3 hours at 4°C, a precipitate of viral particles was recovered by centrifugation (6000 × g for 20 min at 4°C), redisolved in phage buffer **(**20 mM MgCl_2_, 10 mM Tris-HCl pH 7.5, 130 mM ammonium acetate) and kept at 4°C. Serial dilutions of the viral suspension were used to determine the number of infective phages (lytic plaque assays); and the amount of viral particles containing cosmids (transduction of ampicillin resistance).

### Purification of P4 viral particles and isolation of knotted DNA

Viral particles redisolved in phage buffer were banded by cesium chloride centrifugation (33% w/v CsCl at 24°C) in a NVT65 rotor for 14 h at 45.000 rpm. Banded phages containing a cosmid molecule or P4 DNA were pulled out from the tube and extensively dialyzed against P buffer. DNA was extracted twice with phenol, once with phenol/chloroform, precipitated with ethanol, and resuspended in TE buffer (Tris-HCl 10 mM pH 8, EDTA 1 mM) to a concentration about 1 mg/ml. Typically, over 250 μg of cosmid DNA were obtained from the phages amplified in a 1000 ml bacterial culture.

### Unknotting of DNA by yeast topoisomerase II

Yeast topoisomerase II was purified from *S. cerevisiae *strain BCY123 harbouring the topoisomerase II expression plasmid YEpTOP2GAL1 as previously described [34]. DNA unknotting was carried out in 50 μl reaction volumes, containing 50 mM Tris-HCl pH 8, 1 mM EDTA, 150 mM KCl, 8 mM MgCl_2_, 7 mM 2-mercaptoethanol, 100 μg/ml bovine serum albumin, 2 μg of knotted DNA and 20 ng of topoisomerase II. Reactions were started by the addition of ATP to 1 mM. Following 10 min incubation at 30°C, reactions were stopped by the addition of EDTA to 25 mM.

### Electrophoretic analysis and quantification of knotted DNA

Purified DNA was analyzed by one-dimensional or two-dimensional gel electrophoresis as previously described [30]. Agarose gel slabs were equilibrated with TBE buffer (100 mM Tris-borate pH 8.3, 2 mM EDTA) and DNA samples were run at the voltages specified in figure legends. Gels were stained with ethidium bromide and DNA bands quantified with a Fluor-S Multimager system. Where indicated, gels were blotted to a nylon membrane and DNA radio-probed and quantified with a Phosphor-Imager system. Packaging efficiency of cosmids was calculated as the amount of cosmid detected after unknotting by topoisomerase II relative to the total amount of DNA extracted from viral particles. Knotting probability of the packaged cosmids was calculated as the fraction of unknotted cosmid gained upon the unknotting reaction by topoisomerase II.

## Authors' contributions

JR conceived the study, coordinated it, and wrote the paper. ST designed and performed the experimental work. Both authors have read and approved the final version of the manuscript.
